# Dr. Angus Fraser

**Published:** 1912-04-27

**Authors:** 


### Obituary.

Dr. Angus Fraser, consulting physician to the Royal Infirmary, Aberdeen, has died at the age of seventy-four. Born and educated at Aberdeen, he took the M.D. and C.M. of his native University in 1862, and after some study abroad began to practise there. In 1871 Dr. Fraser was appointed physician to the Royal Infirmary, and in course of time became a consulting physician. His connection with this institution was coincident with the successful accomplishment of many administrative structural and other reforms, in which Dr. Fraser can claim a a large share. The University of Aberdeen bestowed on him in 1901 the degree of LL.D., and for some years he was the elected representative of the University on the General Medical Council.

